# Irritation and Inflammation of the Peritoneum

**Published:** 1834

**Authors:** Daniel Drake


### WESTERN JOURNAL

OF THE

### MEDICAL AND PHYSICAL SCIENCES.

JANUARY, FEBRUARY, AND MARCH, 1834.

### Essays and Cases.

### Art. I.—Irritation and Inflammation of the Peritoneum.

*Cases of Irritation and Inflammation of the Peritoneum, from the impress of Foreign Matter; with general remarks on the Pathology and Treatment of Acute Intestinal Diseases.* By Daniel Drake, M. D.

Read before the Cincinnati Medical Society.

The painful affections of the Alimentary Canal, below the diaphragm, which, left to themselves, or resisting medical treatment, prove speedily fatal, are seated in one or more of three different tissues—the mucous, the muscular, and the serous.

In a majority of cases, they begin in one and extend to the others. Thus, when a large dose of tartar emetic or corrosive sublimate is received into the stomach, an irritation is instantly established in the mucous membrane, which throws the muscular into unwonted and irregular contractions, expelling the contents of the tube, both upwards and downwards, and attended with excruciating pain. The irritation is speedily transmuted into inflammation, but this is followed by no abatement of the spasms of the middle or muscular tunic, which, in most cases, are sustained equally by nervous, and nervo-vascular or inflammatory irritation. Again, when the serous membrane becomes the seat of irritation, accompanied or not by inflammation, and the offspring of causes acting either directly on i[illegible]directly through some distant part of the body, the muscular tunic is immediately affected. Its regular peristaltic action is, in general, replaced by painful spasms, which sometimes eject the contents of the stomach and upper bowels, but scarcely ever carry downwards the matters contained in the latter. The muscular tunic may, however, be affected before either of the others, although beyond the reach of foreign matters. This results from its sympathy with other parts of the body. Thus, a disgusting smell or taste excites vomiting—fear increases the peristaltic action —a tobacco poultice to the abdomen does both—and cold to the surface of the body originates in it the violent and irregular contractions, which constitute one of the varieties of flatulent colic.

When the peristaltic function is deranged, it occasionally happens, that one portion of the intestine is drawn into another, either upwards or downwards; should the receiving part not compress it, this accident will do no harm; too often, however, a spasmodic action establishes such compression, when the pain is immediately augmented, and an inflammation begins, which may affect one or all the tissues.

From the rank and value of the alimentary canal in the economy of every animal, we might decide, *a priori*, that the morbid states to which I have referred, must speedily involve the whole organism; and such we know, from observation, to be the fact. In the first stages of all these affections, whether commencing in the mucous, the serous, or the muscular tunic, we invariably find the powers of the heart and brain depressed, and exercised in an irregular manner; and sometimes this depression is so great, as to destroy life in a single day or less. In these cases, but little inflammation is developed, and the principal morbid appearances found on dissection, are those which connect themselves with the distribution of the flatus of the bowels. Oftener, however, the patient lives long enough for nervous irritation to pass into nervo-vascular or inflammation, and its consequences are revealed by dissection after death—in most instances by the symptoms during life. In many cases, however, the action of the heart is frequent and *feeble*, and the developement of heat inconsiderable throughout the whole course of the inflammation; effects which arise, perhaps, from the nausea so generally present, and which, when induced by the mildest artificial means, never fails to lower the powers of the heart and brain, and reduce the energy of the calorific function. Hence results the mixed and uncertain diagnosis of these affections. Nothing, indeed, is more difficult, than to decide accurately on the real condition of the diseased parts, till examinations are made after death. Let us enumerate some of the symptoms:

1. *Pain.*—This continues from the beginning till near the termination of the disease, whether inflammation supervenes, or the patient dies from depression merely. The ordinary method, external pressure, employed to determine whether inflammation be present, is no doubt entitled to consideration; but I cannot regard it as infallible, having seen cases where it afforded relief, while the symptoms, the appearance of the drawn blood, and the beneficial effects that followed its abstraction, evidently indicated inflammation; and on the other hand, having found it insupportable, when, from the termination of the case under a course of treatment not adapted to the cure of inflammation, that condition could not have been developed to any decided extent. Still, the general rule is no doubt founded on fact; and the exceptions may hereafter, perhaps, be explained.

It has been said, that as long as inflammation is not present, the patient is restless and tosses himself in bed; but when the abdominal tumefaction becomes great, he will generally maintain one position, whether inflammation exist or not. It has also been thought, that a criterion is furnished by the paroxysmal character of the pain, it being decidedly such when inflammation is not present, and more continuous when it is. But this is a conclusion on which little reliance should be placed, for the pain is generally paroxysmal, as we find it in puerperal peritonitis or hysteritis, and in both cases depends on the same cause—muscular contraction. Whether the patient dies with, or without, inflammation, the pain continues till near the close of life, and ceases only with his expiring sensibilities.

2. *Nausea and Vomiting.*—Both of these symptoms, or the first only, is generally present, and cannot be considered as pathognomonic either of mere irritation or of inflammation.

3. *Constipation.*—This commonly exists throughout, and has no necessary connexion with any thing but an altered muscular function, which we have seen exists as an essential circumstance in every case.

4. *Abdominal Swelling.*—This is perhaps always present, and cannot be said to afford any special indication.

5. *Rigidity and hardness of the Abdominal Parieties.*—This is probably the same in both cases, unless the inflammation extend to the *peritoneum abdominis*, and affect the muscles, when it is always greatest.

6. *Thirst.*—This, likewise, is generally, if not always present; but is more urgent when inflammation has set in.

7. *Initial Chilliness.*—This is perhaps greater in cases which assume an inflammatory character, than the others; but decided rigors are often present in mere nervous irritation.

8. *Febrile Heat.*—When the patient is of a sanguineous temperament and a vigorous constitution, the heat is well sustained, and often excessive after the developement of inflammation; but in opposite temperaments, and feeble constitutions, a natural temperature may consist with decided inflammation.

9. *The Pulse.*—I have already spoken of this. In all cases it is generally small and frequent; but I have seen it preternaturally slow, when there was, and when there was not inflammation; and have more than once known it extremely feeble, while destructive inflammation was rapidly advancing.

10. *Hysterical Symptoms.*—It might be thought, that the presence of these would indicate a state of pure nervous irritation; but they often show themselves when inflammation actually exists, though the more intense that condition, the less violent are those symptoms; because intensity of inflammation indicates a temperament not favourable to hysteria.

11. *The Mode of Fatal Termination.*—The final cessation of the symptoms in death, does not instruct us fully on this point. The closing scene is indeed nearly the same in all cases. Excessive tumefaction of the abdomen, preventing the descent of the diaphragm and the action of the abdominal muscles, consequently, producing great difficulty of respiration, and confining the patient to his back, with his head and shoulders raised; cessation of pain; sinking of the pulse; reduction of heat; and, in general, some degree of delirium, equally presage the fate of the patient, whether he dies without the supervention of inflammation, or, that condition having occurred, it passes on to gangrene or not.

This uniformity of the closing symptoms, when the *post mortem* examinations which follow, disclose in different cases—first, no inflammatory lesion—second, inflammation producing effusions of lymph, serum, or pus—third, the same state, passing into gangrene, deserves to be considered. Let us begin with the last condition. The greater number of those who die from abdominal inflammation, do not exhibit any gangrenous appearances on dissection. Such inflammations, then, may terminate fatally, without running into that state. But the deposites and agglutinations found among the bowels, would not, as observations made on chronic peritonitis demonstrate, prove fatal; it is not then to them, any more than to the gangrene, that we should ascribe the death of the patient. From what then does it result? Manifestly from the lesion of the functions of the entire organism, by the intensity of the morbid condition of the abdominal. They suffer depression and overthrow, the vital forces become exhausted, and the phenomena of life can no longer be emited. The symptoms of the closing scene are not, then, the *consequences* of effusion, or of gangrene, states which may or may not be present; but of the pathological condition common to all fatal cases, whether inflammation happens to supervene or not. Hence we come to understand, how three patients, who might, after death from abdominal disease, exhibit respectively the effects of irritation, effusion, and gangrene, may, in the close of life, present entire uniformity of symptoms—abdominal inflation, abatement of pain, obtuseness of sensibility, reduction of heat, and vacillation of pulse. These are, in fact, the signs of a fatal exhaustion of the vital powers; and whether, before this stage arrives, the morbid action had led to extravasation, or to the death of a portion of any of the tissues, we have no means of judging till after death.

Let us proceed to discuss some of the general principles which should govern us in the treatment of these affections.

1. When the remote cause is a material and palpable irritant from without, introduced upon the mucous or the serous surface, its removal is a *sine qua non*. Unfortunately this is seldom or never practicable, in reference to those which act upon the peritoneum, and can be accomplished but imperfectly, when, having passed the pylorus, they exert themselves upon the intestinal mucous membrane; for although many irritants excite in the muscular tunic an action, that carries them downwards, and leads to their expulsion; there are others which throw the peristaltic function into an irregular and disorderly action, which tends to confine them in the bowels, where, continuing to irritate, they keep up and augment indefinitely the disease of the parieties. We must not confound with these irritants, certain ingesta taken as food, which appear to be the causes of disease, or the ordinary fœcal contents of the lower bowels, seeing that these are, in fact, rendered offensive to the organ in consequence of an altered condition of its vital properties from other causes, without which they would produce none but their ordinary effects. When in perfect health, a man may indulge himself in articles of diet and drink to a liberal extent, both in quality and quantity, and experience no inconvenience, often rather the reverse; for the best health is by no means the offspring of the greatest abstinence, or the discreetest circumspection; and much experience demonstrates, that mere retention of the conterts of the lower bowels, such as occurs from sedentary habits, grief, occupation of mind, or neglect, is not often, or necessarily, a cause of any of the acute diseases we are considering. From causes of this kind constipation often arises, and continues for several days, without any other inconvenience to the patient, than pain or fulness of the head, or acidity of the stomach. It is the *cause* of the costiveness, then, that should fix our attention, not the costiveness itself; and upon this our remedial efforts should be directed.

2. We come, in the second place, to inquire into the means of restoring a healthy peristaltic function, which, if not the cure of the disease, is a sign of its cessation; but, in general, the means which regulate or restore that function are, in fact, the true remedies. Contraction is the office of the muscular membrane of the alimentary canal. When this contraction is irregular, the transmitting function of the tube is inverted or suspended, and while some parts of the canal are morbidly constricted, others are excessively dilated. On the whole there will, in general, be little or no evacuation, except by the mouth. This morbid condition may sometimes be primary, as it respects the abdominal parieties, as when the individual has an attack of flatulent colic from exposure to cold, or experiences a metastasis of rheumatism from the joints. But this spasmodic and irregular action is, in general, the consequence of a disordered condition of one or both of the membranes—mucous and serous, between which the muscular is enclosed. This disorder may be that, which, in the common parlance of the profession, is denominated a nervous irritation, or it may be an inflammation, or the former in one part of the canal, and the latter in another. Whichever it may be, as I have intimated in the first part of this paper, the phenomena presented by the muscular tunic will be nearly the same. The chief difference, perhaps, will be that in the former case the irregular contractions are more wandering, in the latter more fixed.

But let us come to the *methodus medendi*. This, with the people, is quite plain, and equally obvious to many of the profession. As the function of fœcal evacuation is suspended, and as cathartics will quicken that function in health, nothing is more *natural*, and few things more *unscientifick*, than the immediate administration of purgatives. In some cases of the mildest kind, they may, it is true, bring the peristaltic function under their government, which implies the suspension of the morbid, spasmodic action; but in all the more violent and obstinate cases, so far from accomplishing this object, they exert on the canal no specific action, because its sensibilities are changed, and, acting as irritants, they augment the spasmodic action; by increasing the nervous irritation, or the inflammation, according as one or the other may happen, to be the existing morbid action. Let us proceed, then, to consider th emeans by which these disordered conditions of the nervous and the nervo-vascular extremities may be quieted.

3. Of nervous irritation. The means of allaying this, are chiefly those which belong to the class of narcotics—of which opium and tobacco may be cited as the most powerful. Without going into an elaborate inquiry as to the *modus agendi* of narcotics, we may say, as practical men, that they diminish morbid sensibility, and quiet the spasmodic action which springs from it, precisely the effects that we desire; for upon subduing the spasms, the powers of the system soon re-establish a healthy peristaltic function; or if they should not, we may excite it by cathartics; which, the morbid sensibilities of the mucous membrane being appeased, will prove inoffensive and speedily raise in the muscular tunic, their specific excitement. But to produce these desirable effects, they must be exhibited at once in decisive doses; and those, which, like opium and tobacco, have the least of a stimulating, and most of a relaxing and stupefying effect, will always prove most beneficial. I am aware that in cases of this kind, some writers, particularly Dr. Abercrombie, are of opinion, that certain parts of the bowel are paralysed, and therefore distended to a painful and fatal degree, and hence that opium is contra-indicated. But the same gentleman recommends tobacco, which is still more relaxing, and consequently would be more prejudicial. Moreover, whence comes the gas which produces this distension? I cannot doubt that it is the offspring of a morbid action, distinct from that in the muscular tunic; and believe that its generation may be successfully met, in most cases, by the administration of narcotics. As to the contracted parts of the intestine being in a healthy condition, and the expanded in a morbid state, I am disposed to believe the opinion to be the reverse of the fact; for great muscular contraction may suddenly take place in the hollow viscera, as we often see in the urethra, and as suddenly cease; but paralysis, except it depend on disease of the brain, is not often suddenly induced, and never removed with the speed that we observe distended portions of bowel to recover in certain forms of colic. The recovery from paralysis is, indeed, always slow. On the whole, I think it probable, that there is temporary defective contraction of one part of the bowels, by which it fails to protect itself against distension, and excessive contrac tion in another part, throwing the flatus into that which makes least resistance; and that these opposite conditions may change their places, and thus lead to the transfer of the flatulent accumulations from one part to another, giving rise to borborigmi. Now, the defective and the excessive contraction of which I speak, are not distinct and opposing maladies, to be met by opposite means, but resemble the spasmodic diseases of the muscles of locomotion, from irritations of the nervous centre, in which we always observe some muscles to be in a state of relaxation or quiescence, while others are in spasm. How, then, is the case to be met? Evidently with the means of equalizing that mysterious influence, on which the muscular contractions of the bowels in health, may depend, and these are chiefly the narcotics administered in stupefying doses. These medicines, however, will not always succeed in allaying the nervous and muscular irritability, even when inflammation is not present. The system is sometimes insensible to their impress, and little or no effect will come from them. This is apt to be the case in phlegmatic temperaments; and the same condition is often connected with plethora. Whenever this state of things manifests itself, the grand resource of the profession is blood-letting. The *modus operandi* of blood-letting, in this exigency, is by increasing the sensibility and irritability of the system. Nothing does this so effectually as the loss of blood; and nothing, in general, is wanting, but this renovated vital susceptibility, to give immediate and beneficial influence to the narcotic. The value of blood-letting, in these cases, is not always appreciated by the profession; and some of its members even dread the employment of it. But whenever nervous irritation and spasmodic action of the abdominal viscera, suddenly induced, resist the exhibition of narcotic medicines, I believe blood-letting not only safe, whatever may be the temperament of the patient, but imperatively required. To be salutary, however, it should be preceded and followed by liberal doses of opium, or some of its preparations.

4. Of inflammation. All inflammations are preceded by nervous irritation, with depression of vital power, in the parts where they arise. In some cases of intestinal disease the reduction and perversion of the vital power, are so great, as we have already seen, that they destroy life, before a sufficient time has elapsed for inflammation to be developed. These cases, however, are comparatively few in number; and whenever the attack extends beyond a single day, inflammation should be apprehended. The nature of the remote cause, the temperament of the patient, and the previous condition of his system, will determine, in a great degree, the time and intensity of the inflammatory symptoms. I have no doubt, that inflammation may be developed in some parts, while others remain in a state of simple nervous irritation. Of the difficulty of deciding on the actual condition of the parts, in many of these cases, I have already spoken. Most of those which prove fatal, are attended with more or less inflammation, and from the transient character of violent nervous irritations, I have no doubt that more or less inflammation is always developed in protracted cases, although the vestiges of it may not always be found in the mucous and serous membranes; for, as Dr. Abercrombie has admitted, it may attack the intervening muscular tunic. That gentleman has reported several deaths from colic or ileus, in which he informs us, no traces of inflammation were observed; but the general experience of the profession undoubtedly is, that in most of these cases, inflammation sets in before the patient dies. The following are the words of the writer, just quoted, in reference to this matter:—

"The usual progress of the disease in fatal cases, is into inflammation and its consequences; and we have seen it fatal while the inflammation was in various stages of its progress, from a recent tinge of redness, to extensive gangrene." *Page* 160.

In reference to the *post mortem* appearances in colic, Dr. Thomas expresses himself in these words—

"When the disease proves mortal, the usual appearances to be observed on dissection, are inflammation on the surface of the intestines, distension and irregular contraction of some parts of the tube." *Page* 368.

Of the morbid appearances after death, from the variety of colic named ileus or volvolus, Hoffman expresses himself in these terms:—

"The ileum is found convoluted, in some parts inflamed and spacelated, and the part above the inflammation remarkably distended with flatulencies." *Vol.* I. *p.* 500.

I shall not extend these authorities. My chief object is to show, that those forms of intestinal disease, which begin as nervous irritations and spasms merely, are liable in their progress to become inflammatory, and before they prove fatal, are generally attended with that condition. These make but a part of the intestinal affections which we are reviewing; but, with respect to the remainder, there can be little doubt of the existence of inflammation from an early period.

Now, this inflammation, whether mild or intense, circumscribed or diffused, scarcely ever fails to derange the peristaltic function, producing, or maintaining constipation, and rendering the bowels wholly insensible to the action of cathartics. Being a proximate cause, then, of the irregularities in the peristaltic function, it must be removed or abated, before that function can be restored. But is this important rule of practice always observed? I fear it is not. I have violated it myself, and seen it still oftener violated by others. I am persuaded, from much experience, that it is far safer to bleed, when bleeding is unnecessary, than to omit it when required. To expect to overcome the constipation, and relieve the patient from pain and distention, when the bowel is inflamed even in a slight degree, is a crude, painful and unscientific proceeding; which, moreover, has cost many patients their lives. The substitution of active purges for blood-letting, in these cases, is, therefore, not merely a negative, but a positive injury, for while the latter would do good, the former are actually of an irritating nature and prejudicial.

Venesection is, generally, the proper kind of blood-letting for such patients; but where the inflammation is slight, or the system of the patient has been previously enfeebled, or phlebotomy has been carried to a considerable extent, local blood-letting by cups or leeches, is preferable, to further venesection, and often produces the most admirable effects—abating the inflammation, without further exhausting the patient, and frequently exciting the bowels into action beyond the use of any other means.

5. In the fifth and last place, I come to speak of the secretions. These are, generally, deranged or suppressed, in all the forms of violent intestinal disease, which we are considering. The restoration of them is an object not to be lost sight of and should, if possible, be accomplished before evacuation from the intestinal canal is attempted. For this purpose in part, we give opium and draw blood, as we can have little hope of restoring the suspended secretions, while great nervous irritation or inflammation is present. These morbid states being abated, we may act successfully upon the liver with large doses of calomel; and on the mucous membrane of the bowels, with saline and other gentle and hydrogogue cathartics, such as castor oil, sulphate of magnesia or soda, super tartrate of potash, and the tartrate of antimony. The secretory functions being in some degree restored, the remaining inflammation will be rapidly reduced, and copious purging readily effected, even by emollient injections.

Such are the various means by which the violent and painful affections, known under the names of colic, ileus, enteritis, intus-susception, and peritonitis, are to be met. The ultimate object in the treatment of the whole, is the restoration of the secretions and the peristaltic function, effects which we too often seek, in the first instance, to effect with powerful cathartics, when, in reality, they should, as a general rule, be the last items in the series of remedial agents.

It is not difficult to conceive of the observations which first suggested this practice. It must have been observed long ago, that when copious purgings took place in these maladies, the patient generally recovered—when it did not, he died. On this fact seems to have rested the rule which prescribed purgatives, with a resort to the more drastic and to larger doses, as the costiveness proved more obstinate,—the very reverse of what is safe and successful—for the greater the torpor of the bowels, the more intense is the irritation, nervous or vascular, which produces it, and, consequently, the greater the necessity of withholding those drastics, which might convert irritation into inflammation, or aggravate the latter, if it already existed.

But although it is easy to understand how our predecessors and the people, came, by confounding cause and effect, to believe that the purging was the main step in the cure; it is difficult to comprehend, how, in the present improved state of the science, this wrong association of ideas should continue, and exercise an influence, so decisive as it sometimes does, in the practice of what are called regular bred physicians. For the honor of the profession, I am happy to say, that the error is far from being universal, and that there are not a few of its members who direct their efforts upon the morbid condition, which produces the irregular or suspended peristaltic function, and by subduing it, enable the bowels, either spontaneously, or by the aid of gentle cathartics, to resume their action.

Having gone through an inquiry—far from a perfect one, I admit—into the pathology and therapeutics of several kindred affections of the intestinal canal, I propose now to say something in detail on one of them—that of the peritoneum occasioned by the action on its surface of foreign matters.

I need not remind the members of this society, of the general anatomical and physiological characters of this membrane. Closely enveloping the intestines, it is, at all times, quite contiguous to a surface, on which rest a great variety of gross matters, but still it has no capacity for bearing their impress; and a copious experience has shown, that when any of them, or foreign matters of any other sort, enter its cavity, effects, both local and constitutional, of the most alarming kind, are immediately developed. A law of the organism has been violated, and the consequence, in general, is death. Doubtless, many have died from this cause, without its having been suspected; but cases enough are on record to furnish us with the symptomatology and morbid anatomy of these varieties of peritoneal disease; if not with the means of curing them. I shall proceed to enumerate the principal:

1. Extravasations of blood, from mechanical ruptures of the liver or spleen.

2. Discharges of pus, chiefly from abscesses of the liver.

3. Escape of the contents of the gravid uterus, by a rupture of that organ.

4. Effusions of urine, from the bursting of the bladder.

5. Escape of the contents of the stomach and bowels, from wounds, rupture, or ulceration.

6. Escape of the cystic bile from the same lesions of the gall bladder or its duct.

The effusion of blood into the cavity of the peritoneum, is rarely attended with much disturbance of its functions, but occasionally inflammation supervenes.

That of pus is not quite so harmless, as it is more difficult to absorb, and more irritating.

The escape of the contents of the gravid uterus, is, generally, fatal in a few hours. Vomiting takes place immediately, the heart fails in its powers, the patient becomes cold, and expires. In these cases we must add the effects of the laceration of the uterus, to those of the impress of the fœtus upon the peritoneum. Between the two, the patient commonly dies before inflammation is established.

The effusion of urine into the peritoneal sack, is a grave and fatal accident.

It has never happened to me to meet with a single case of any of these accidents; and I conclude, therefore, that they are of uncommon occurrence, and I shall dismiss them without further notice.

The effusion of the contents of the stomach, the bowels, and the gall bladder—particularly of the bowels—is a much more frequent event; and although, perhaps, always fatal, it deserves on various accounts, to fix the attention of the profession.

This effusion may be the consequence, 1, of wounds; 2, of contusions; 3, in reference to the gall bladder, of a puncture by the lancet when the organ is distended, simulates the form and other characters of a hepatic abscess; 4, in reference to the same organ, by distention and rupture; 5, of inflammation and softening of the stomach and bowels; 6, of ulceration and absorption.

When the bile only escapes into the cavity of the peritoneum, we may presume that the consequences are less urgent, than when the contents of the stomach or bowels, are effused, as they comprehend various crude matters, in connexion with that fluid.

The first effect of the entrance and spread of the contents of the primæviæ, or the gall bladder, upon the surface of the peritoneum, must be those of a gross foreign matter on a living surface, not previously subjected to that kind of action, nor indeed prepared to sustain it. Hence a series of morbid actions immediately commences. The symptoms which express the first part of this train are irritative; those which characterize the second, when the first do not prove fatal, are inflammatory. Let us enumerate the principal—excruciating pain, suddenly induced, and often beginning in a particular part; chilliness; vomiting; a small, feeble, and frequent pulse; paleness; alarm; muscular debility, and sometimes death in a few hours, from prostration of the vital functions; symptoms not unlike those from extensive burns and scalds, great lacerated wounds, or energetic acrid poisons received in large quantities into the stomach or a wound. If the patient should not fall an immediate victim to this irritation, other symptoms begin to blend themselves with those just enumerated. Costiveness is found to supervene, the abdomen begins to swell, and become painful on pressure; the patient complains of thirst; the pulse still continuing small and frequent, becomes wiry; the diaphragm and abdominal muscles act with difficulty, giving rise to embarrassed respiration; the secretion of urine is suspended—symptoms which sufficiently indicate that inflammation has supervened upon irritation.

The foreign matter, however, still remaining in contact with the peritoneum, depresses the vital powers of the heart and brain, and however acute and rapidly disorganizing the inflammation may be, those governing organs never recover their tone, and the signs of debility and irritation in the sanguineous and nervous systems continue, and are blended with those of inflammation.

The proportions in which these two classes of symptoms occur in the same case, will depend on temperament, and the rigor of the constitution. When both favor the development of inflammation, it will take place speedily and extensively, though without the constitutional signs of an intense inflammatory condition; but, on the other hand, if the patient be of a nervous or phlegmatic temperament, or prone to hysterical irritation, the degree of inflammation, even when death does not take place for several days, will be inconsiderable. I have now before me, notes of four cases, occurring in this city within the last six years. The symptoms of all were nearly the same; in three of the patients the ravages of inflammation were found after death; in the other, none were *said* to be present, but still, it had existed, for the blood drawn 24 hours before death was sizy. The three former cases were from rupture of the bowels, the latter from that of the gall bladder; and here the question presents itself, whether the impress of the bile upon the peritoneum, is not of that kind which developes great nervous irritation, rather than inflammation. I am disposed to think that this is the fact, not only from the history of this case, compared with the others, but from the circumstance that in jaundice, when the elements of the bile are circulating throughout the system, the pulse is generally weak, the nervous system depressed and irritable, and inflammatory symptoms often absent, or feebly declared. Any acute inflammation of the liver, does not admit of the great loss of blood, like inflammation of the lungs or bowels, and I

can perceive no reason for the difference, but the absorption or retention of bile in the blood, in the former case; and its regular excretion, in the latter.

To whatever extent inflammation may be developed in these patients, and whether it terminate in gangrene, or the patient die from prostration and dyspnoæ previously to that disorganization, the final symptoms are nearly, or identically, the same—great distention of the abdomen; cessation of pain; sinking of the heat of the surface; pallid, or purplish face; shrunken features; extreme difficulty of breathing; a wavering, and, at length, imperceptible pulse; clammy sweats; and a fixed position on the back.

Of the treatment of these cases, all of which, perhaps, are necessarily fatal, I need say but little. Could we be assured that n rupture had taken place, nothing need be attempted, but the exhibition of narcotics; but as this can seldom or never be known, it is our duty to prescribe according to the symptoms, on the theory of irritation, spasm, or inflammation from some common cause, for which I will refer to what has been already said, in a former part of this paper. In regard to blood-letting in these accidents, it was either injurious or useless in every case in which I have seen it employed. But when we do not know of the presence of foreign matter in the cavity of the peritoneum; and tension, pain, and tenderness of the abdomen, are present; what can justify the postponement of venesection? The lancet, moreover, is an instrument of diagnosis in these affections, for if there be extraneous matter upon the peritoneum, the pulse will sink, instead of rising, under its use.

The following observations of Dr. Graves and Dr. Stokes, on this subject, are so judicious, that I trust the Society will excuse me for reading them from Dr. Hays' abridgement of the Medico-Chirurgical Transactions, pp. 374–7.

"The foregoing cases of peritonitis from perforation of the intestine, having occurred within a short space of time, gave us a good opportunity of studying the disease. In most of them the perforation was owing to an ulceration of the mucous follicles of the ileum, preceded by a process of irritation for a greater or lesser space of time. Thus we have seen the disease occurring in cases of gastric fever, and in diarrhœa, either idiopathic or induced by the use of purgative medicine; circumstances in which the mucous system is called into morbid action. Further, in all our cases, with but one exception, we have found near to the perforation, numerous marks of irritation in the muciparous system. Some of the follicles were enlarged, others softened down and forming ulcers, the bases of which were frequently formed by the serous membrane.

The symptoms in most of our cases were very similar. The sudden appearance of the peritonitic symptoms, and the rapid sinking of the powers of life, pointed out the occurrence of the perforation in the majority of cases. In fact, the peritonitis from perforation differs in this from idiopathic, that the patient is brought in a very short time to the last stage of the disease. An extent of injury takes place in an hour, which in the common peritonitis would perhaps take days to produce.

In one case, however, Pilkington, the disease was slow and latent. Here the only evidence of any abdominal affection was the recurrence of a slight tenderness of the epigastrium, which was in every instance removed by the application of a few leeches. We have heard of another case which occurred in this town, where, after a long fever without local symptoms, the intestine was found perforated in numerous places. In Pilkington's case, it is to be remarked, that no fecal matter escaped into the cavity of the peritoneum. The ulcerative process was evidently slow, and the abdominal exudation prevented the escape of the intestinal contents.

In the history, then, of this form of peritonitis, it is of the utmost importance to recollect, that it may take place in a very latent manner: fatal peritonitis may be going on unsuspected by the physician. This is to be particularly recollected when there is an epidemic tendency to affections of the intestinal tube, ulceration of the mucous follicles is more frequently observed to occur in a latent form than the peritonitis which results from it. Thus, we have known it to be found to a very great extent, and involving numerous patches of glands in the ileum of an athletic man killed by accident. During the late epidemic we only observed a single case of fever out of several thousand, where perforation took place, while during the last year this occurrence has been frequent in Dublin.

In the case of Michael Gill, the cessation of pain and tenderness was so remarkable, that we suspected the occurrence of gangrene; this was, however, not borne out by the dissection. Sudden cessation of pain and tenderness at the close of a violent abdominal inflammation, is then not a certain indication of gangrene: it may be produced by the depressed state of sensibility, brought about by the violence and extent of the disease.

We are of opinion, and we beg to call the attention of our readers to this circumstance, that the principle of treatment in these desperate cases is not yet fully understood. Are they to be treated like cases of idiopathic peritonitis? This is a question of the highest practical importance. We shall examine it briefly.

It appears to us that the antiphlogistic method is not so applicable as in the simpler form of this disease. In the first place the perforation generally occurs at an advanced period of some other disease, when the constitution is less capable of supporting any loss of blood. In the second place, the disease, as we stated before, runs its course with such rapidity, that in a comparatively short space of time it brings the patient into the last stage. Now it is at this time the physician is most frequently called on to interfere, and as far as our observation has gone, the antiphlogistic plan only accelerates the fatal termination.

We know that nature makes an attempt to cure, and the question is, How are we to prolong life so that organization of the false membrane may take place? Is it by drawing blood after the peritonitis has come nearly to its last stage, when the powers of the nervous system are in the greatest state of depression? We apprehend not. Bleeding in peritonitis is useful in preventing the increase or extension of the disease; but in these cases the affection is *at once* extensive and severe.

These considerations make us submit that general, or even perhaps local bleeding, is a mistaken practice in this disease, and we are borne out by experience of cases somewhat similar, in recommending a different plan. In the first place, the antiphlogistic plan has failed in all our cases; this alone is an argument against it. Secondly, we know that certain cases of peritonitis, where the powers of life are greatly sunk, may recover under the exhibition of wine and opium, when blood-letting would be destructive. Thirdly, the exhibition of opium in large doses has succeeded in our hands in checking peritonitis, where the powers of the system were so much depressed as to make us avoid the atiphlogistic plan. In the case of Donegan, the use of this remedy was attended with the most singular benefit, and two cases have occurred to us where violent peritoneal inflammation succeeded to tapping for ascites in exhausted subjects; in both, although in a state apparently desperate, the disease was subdued by opium, without the use of either the lancet or leeches. *It is now nine years since this practice was introduced into the Meath Hospital.* In speaking of hepatic abscess, we have adduced another example of cure of general peritonitis, supervening on the opening of the abscess, attributable to the use of opium and wine; here the peritonitis was universal and its cure perfect, as was shewn by dissection.

We would then recommend the use of opium *in large doses* in cases of peritonitis from perforation. If the lancet is to be used, it should be only at the very outset of the disease, and even then its utility appears to us very problematical."

I shall now proceed to illustrate what has been advanced, on the symptoms, pathology, and morbid anatomy, of the peritoneal disease consequent on ruptures of the stomach, bowels, and gall bladder, by reading a number of selected and original cases.

### I. Escape of the Contents of the Stomach.

1. *From Perforation.*—A gentleman, aged about 60, in the year 1825, had for a considerable time suffered from complaints in his stomach. He had occasional pain, but it was not severe; his more prominent symptoms were an intense feeling of Pyrosis, and occasional vomiting. He was often obliged to leave the table suddenly during meals from attacks of this kind, in which he chiefly brought up small quantities of an extremely acrid fluid. He became much emaciated, and had every appearance of extensive organic disease, though none could be discovered on examination. He required to be kept upon the most cautiously regulated diet; and after continuing for some months in a state from which he was not expected to recover, he gradually got into his former good health, and his stomach entirely recovered its healthy functions. He had at various times, however, slight threatenings of his former symptoms, and required to live with great caution; but he was full in flesh, and his general health was excellent. About a fortnight before his death, he had one of those slighter attacks, which affected him chiefly with a distressing feeling of Pyrosis, impaired appetite, and occasional vomiting. On account of these symptoms he was keeping the house, though able to attend to the affairs of an extensive business, until Saturday evening, 3d February, 1827, when he was suddenly seized with excruciating pain in the pit of the stomach, accompanied by some vomiting, coldness of the body, and a small frequent pulse. From the moment of this attack, nothing that was done afforded the least relief. He continued in the most violent and unceasing pain through the night and through the following day; The whole abdomen became distended and tender, with sinking of the vital powers, and he died on Sunday night, about 30 hours after the attack.

*Inspection.*—On the posterior surface of the stomach near the Pyloric extremity, there was a space rather larger than a shilling, where the substance of the stomach was entirely destroyed; but the margin of the opening adhered all around very closely to the surface of the liver, which thus preserved the continuity of the part. Below this portion, and very near the Pylorus, there was an ulcer on the mucous membrane, smaller than a sixpence, and through this a perforation of the coats had taken place of such an extent as would have transmitted a full sized quill. Through this opening the contents of the stomach had escaped into the cavity of the peritoneum, where there were exhibited the usual marks of extensive but recent peritonitis. Except the two spots now referred to, the stomach was perfectly healthy.

*Abercrombie, pp.* 58--9.

2. *From a fissure of the Pylorus.**—In the afternoon of the 14th of April, 1828, I was called to see E. P., aged 38 years, by occupation a cordwainer. He was said to labor under colic; and to have been

* This and another case have been already published in this Journal, and it is not without some misgivings that the *Editor* here inserts them. His apology is, that they were inserted in early numbers of the work, that it has acquired many new subscribers, and that the paper read before the Society would be defective without them.

attacked suddenly and violently. I found him sitting up, with a pale and cool skin, a feeble pulse, beating not more than 48 strokes in a minute, and complaining of excruciating spasmodic pain in the abdomen, the paroxysms of which were followed by incomplete fainting fits. Regarding it as an attack of flatulent colic, I, immediately directed a hot foot-bath; and began the preparation of a large dose of opium and calomel. Before it was ready for admnistration, he was seized with retchings, and drank a glass of tepid water. Slight vomiting occurred, with agonizing pain, which was followed by a degree of syncope that bordered on dissolution. As soon as he revived the portion of opium and calomel was administered, together with a quantity of paregoric. Stimulating, cathartic enemas were then employed; but they produced no fæcal evacuation, and, indeed, were themselves but partially discharged. About 10 o'clock I left him for the night, in the care of my young friend Dr. McDowell. The Calomel and opium were repeated; laudanum was liberally administered; a dose of castor oil was given, and the injections were repeated, in the course of the night; but nothing more than palliation followed. The next morning I found him thirsty, in pain and drowsy, with his abdomen distended, especially *in epigastrio*, and tender on pressure. His pulse was feeble, exceedingly variable in its rythm, at no time more than 60 in a minute, and frequently down to 46. It was obvious that peritonitis had supervened, and I bled him. The blood was sizy; but his pulse fell instead of rising in its energies; no alvine evacuation ensued; nor were the pain and swelling diminished. Calomel was now administered in large and repeated portions, with occasional doses of sulphate of magnesia and castor oil; the injections were repeated, and the abdomen was blistered. In the evening the symptoms were mitigated, and a small quantity of feculent matter was discharged. During the night a gentle salivation came on; he rested badly; had occasional retchings, in which he threw up but little; and the next morning was worse, than I had left him in the evening. The epigastric tension, and soreness on pressure, especially had increased. The pulse remained nearly the same in frequency, but was still weaker and smaller. The case now gave me equal embarrassment and apprehension; for it exhibited unequivocal evidences of abdominal inflammation, combined with an exhaustion of the vital powers, which was as manifestly occasioned by something else, than the peritonitis, which had been developed. I could not doubt that a structural lesion existed; but of what kind it seemed impossible to determine. Examination was made for hernia—ventral, inguinal and femoral—but none existed; and I was led to conjecture that intussusceptio had taken place. I desired a consultation, and, in the afternoon, Dr. Pierson was called in. He was struck with the obvious signs of peritoneal inflammation; and the patient was again bled more copious than before. The pulse sunk during the operation, and the blood was not sizy. The abdominal tumefaction and agony increased; his pulse became imperceptible, and toward morning he expired; about 60 hours from the commencement of the attack.

***Examination of the Abdomen.***—Leave being granted to inspect the abdomen, it was done the evening after his death. The distention was very considerable, and upon penetrating the cavity of the peritoneum, a great quantity of offensive gas escaped. On making an extended crucial incision, that membrane was found to be inflamed, and to have thrown out a quantity of coagulable lymph, with some admixture of pus. It was slightly agglutinated to the bowels; and these were extensively adherent to each other. The whole were immersed in a turbid liquor, of a yellowish colour, which emitted the smell of turpentine, and exhibited no inconsiderable quantity of oil floating on its surface. A large worm (lumbricoides) was crawling over the bowels. After sponging out between two and three quarts of fluid, (the quantity extravasated) we found the outer tunic of all the anterior and upper convolutions of the intestines, great and small, in a state of inflammation. The omentum was in the same condition. The spleen was large, tender and so much inflamed, that its peritoneal covering peeled off. The liver was but little affected. The gall bladder was distended with healthy bile. The urinary bladder was empty and uninflamed. On examining the intestines, from the rectum to the stomach, no lesion was found, till we reached the pylorus; where the cause of all the mischief was discovered. *It was ruptured more than half way round, and the fissure exhibited the appearance of a clean cut.* Through this, the contents of the stomach and duodenum had escaped into the cavity of the peritoneum. On slitting open the bowels and stomach, the mucous membrane of the former was healthy, and the pyloric extremity of the stomach was equally healthy, on both sides. The mucous coat of its great extremity was considerably, but not deeply inflamed. No ulceration existed any where; nor had any part suffered gangrene. The closest inspection of the pylorus, did not enable us to detect any organic change, that could account for its laceration.—*West. Journal, vol.* 2, *pp.* 127-9

### II. Escape of the Contents of the Small Intestines.

*From Perforation of the Ileum.*—A girl, aged 15, (12th May, 1818) had fever, with pneumonic symptoms; was bled with relief; the fever subsided gradually, and on the 19th she was considered as well. On the 20th, at night, she complained of some pain of the belly, which soon went off, and through the night she felt no uneasiness. On the 21st, had violent pain and tenderness of the abdomen, with some vomiting; pulse frequent. Took an opiate, and afterwards some purgative medicine; the vomiting subsided after the opiate; the pain also was much alleviated, and was only complained of upon pressure. The purgative did not operate during the day, but operated freely in the night four or five times. I saw her for the first time on the morning of the 22d, and found her moribund; the pulse not to be counted from its frequency; features collapsed; belly tympanitic. She died in less than an hour after the visit.

*Inspection.*—On the surface of the bowels on many places, especially on the ileum, there was peritonitis with deposition of false membrane. On the inner surface of the ileum, near the caput coli, there was an inflamed portion, in the centre of which, there was a white spot the size of a shilling; and, in the centre of this spot, a round perforating aperture, which transmitted a quill; the edges of it were rounded, and a little thickened. Much fluid feces and gas had escaped into the cavity of the peritoneum, and the bowels were not distended; there were in some places a few livid spots, but no gangrene.—*Abercrombie, p.* 179.

2. *From Perforation of the Ileum.*—Patrick Kane, aged 46, a laborer, was admitted on the 2d of October, 1828, laboring under severe symptoms of peritonitis. About a year previously this man had contracted ague, for which he took bark, which stopped the rigors, but he never recovered his appetite, and labored under continual thirst, with occasional soreness in the region of the stomach, and yellow slimy dejections. Twelve days before his admission he was exposed to cold, when he was attacked with shivering, followed by total anorexia, vomiting, and severe pain across the umbilical region. These symptoms were succeeded by a bilious diarrhœa, which continued for ten days, when suddenly the pain became intense, the diarrhœa ceased, and he found himself unable to pass his urine; the pain was general over the abdomen, and was particularly severe in the epigastric and umbilical regions; swelling of the abdomen appeared immediately after. The following was the report of his state on the day of admission. The belly is swelled, tympanitic, and extremely tender; pulse 88, small and compressible; respirations hurried, with occasional moaning. He passed his urine to-day, and has had some vomiting; no stool. Yesterday had occasional singultus; *leeches were applied to the abdomen; tobacco and purgative injections.*

October 2d. Pulse somewhat fuller; pain rather less; no evacuation by stool; copious vomiting of a brown fluid; venesection to ℥xvi. three grains of calomel every second hour.

3d. A copious purging came on at midnight of natural colored fæces; vomiting continued. There is no pain or tenderness of the belly; pulse imperceptible; countenance sunk; singultus. Death at noon.

*Dissection, fourteen hours after Death.*—Belly tense and tympanitic. On opening the peritoneal cavity a quantity of fœtid air escaped, and the sac was found to contain much yellow fluid, mixed with fecal matter and flakes of lymph. The peritoneum covering the liver and abdominal parietes, presented a thin coating of false membrane, easily detached by the scalpel. The small intestines were of a livid color, and covered in many places by coagulable lymph; the lower portion of the ileum adhered strongly to the bladder by very consistent lymph. On its being detached, we found a large perforation of the intestine corresponding to the fundus of the bladder; it was caused by a follicular ulceration occurring about twelve inches from the ilio-cæcal valve. In this part of the gut we found numerous ulcerations of the same kind, of various depths, but no other perforation.

*Med. Chir. Trans. pp.* 368–9.

3. *From Laceration of the Jejunum.*—December 1st, 1827—John McDonald, coachman of Mr. P. B. of this city, aged about 22 years, received a kick from one of the carriage horses, standing in the stable. The accident happened at 3 o'clock, P. M. and the unfortunate man expired between 8 and 9 o'clock in the evening of the next day, or in about 30 hours. From the complaints and statements of the patient, the shod foot of the horse struck him on the left and front part of the abdomen, between the diaphragm and the crest of the ileum; but he could not indicate the spot, and no appearance of the integuments enabled me to identify it. He was able to walk from the stable to the house after the accident; but immediately laid down and complained excessively of abdominal pain, and exhibited a kind of hysterical restlessness. His pulse was considerably increased in frequency, but retained as much force and fulness, as can be associated with that condition. As he had not dined, I presumed his stomach to be comparatively empty; and, therefore, not liable to rupture; the constitutional symptoms, moreover, seemed not to warrant the opinion that such an accident had occurred; and viewing it, though with some doubts, as a case of nervous irritation, I directed 30 drops of laudanum internally, with towels dipped in *hot* water to the abdomen.

In visiting him at night, I found him still in great pain and inquietude. His pulse nearly the same; and his abdomen considerably swelled, tense, and painful to the touch throughout its whole extent. Still more perplexed than before, as to the pathology of his disease, I took twenty ounces of blood. It produced a strong disposition to syncope, but no alleviation of his distress. Ordering two ounces of sulphate of Magnesia in divided doses, I left him for the night.

Next morning, I found him much worse. He had slept none; the pain continued; he had vomited several times; no fæcal or urinary evacuation had taken place, though being thirsty he had drank copiously all night; the abdominal swelling had increased; and his pulse, more frequent than in the evening, had lost much of its volume and firmness. Having seen great benefit from large doses of calomel in peritoneal inflammation attended with feeble pulse, and incapable of comprehending more of the case, than that inflammation was present, I administered 30 grains of calomel.

At 12 o'clock all the symptoms were greatly aggravated. The nurse reported that he had discharged nearly half a pint of urine tinctured with blood; but as he complained of great pain above the symphysis pubis, labored under priapism, and had drank freely, I determined on introducing the catheter; not a drop of urine or blood, however, was evacuated.

I was now convinced that a blood vessel had been ruptured, and internal hæmorrhage existed; or that a rupture of some portion of the alimentary canal had happened; or that extensive inflammation had been suddenly developed, and would speedily terminate in gangrene. Regarding either of the two former læsions as almost inevitably fatal, I thought only of prescribing for the third; and censuring myself for not having drawn blood more copiously in the night, I resolved to make, at least, nominal amends for the supposed omission, and opened a vein. Not more than five or six ounces would flow, and at the end of the operation, his pulse was nearly imperceptible, and his skin leaden, clammy and cool. Directed ol. recini ℥ij, cathartic enemata, a blister to the abdomen, and a hot foot-bath.

At seven in the evening, his pulse was imperceptible at the wrist, and extremely feeble in the carotid; his respiration greatly embarrassed, and his mind wandering; all the symptoms, in short, portended approaching dissolution, though he was still able to move about in bed, and sit up without support. In less than two hours afterwards he expired.

*Appearances on Dissection.*—Permission to examine the body having been granted, the dissection was executed the next day at 11 o'clock, fifteen hours after death.

The abdomen was greatly distended. The skin exhibited no signs or effects of contusion. We were struck with the induration (almost corneous) where the blister had been applied, although it seemed, while he was alive, to have produced no effect. This change of texture, which, doubtless, had no connexion with his malady, would seem not to be of easy explanation.

On dissecting up the integuments, we found an extravasation of blood upon the external oblique muscle of the left side, and between that and the internal, near the junction of their tendinons and fleshy portions betwixt the anterior superior spinous process of the ileum and the umbilicus. This determined the spot which had received the blow. On puncturing the peritoneoum, a great quantity of gas escaped. On laying it open, there flowed and was sponged out, more than two quarts of turbid fæculent yellowish fluid, without any blood. Indeed, no hæmorrhage had existed; but that a rupture of the stomach or bowels had taken place, was sufficiently obvious. The peritoneum, both of the parieties and intestines, every where exhibited signs of inflammation. The omentum had been much inflamed. The intestines, generally, were agglutinated together, and to the walls of the abdomen. There had, indeed, been universal peritonitis, but death occurred before it had terminated in any fatal organic læsion. The inferior or pelvic portion, exhibited signs of greater inflammation than the epigastric portions, doubtless because the extravasated contents of the intestines, had gravitated to the lower part of the abdomen. The bladder was quite empty, much contracted and inflamed in its outer coat.

Beginning with the rectum, I examined the bowels upwards, to find the rupture. All was sound till I came within eighteen inches of the duodenum, where I found the jejunum cut transversely one-third across. The edges of the wound were lacerated, thickened, somewhat rolled back, and adherent to the outer coat of the bowel upon which they were turned. In another place, close to this, the intestine was nearly cut through. The mucous membrane was but little inflamed. The peritoneal covering of the liver, gall, bladder and spleen, was slightly inflamed. The cellular substance over the second and third lumbar vertebræ was injected with blood, and much thickened. The left psoas muscle was the seat of considerable extravasation—it was swelled, tender, and had some of its fibres torn. It was sufficiently obvious, that the bowels had been forcibly pressed, by some part of the shoe of the horse, against the lumbar vertebræ and thus lacerated. Such an injury could not, *of itself*, occasion speedy death, and the early and fatal termination of this case, can only be referred to the impress on the peritoneum, of the contents of the alimentary canal, escaping through the aperture in the jejunum.—*Western Journal, vol.* 1, *pp.* 550–4.

### III. Escape of the Contents of the Great Intestine.

1. *From Perforation of the Colon.*—Michael Donegan, aged 12, was admited on the 22d of September, 1829. It appeared that ten days previously to his admission, he was attacked with severe pain in the abdomen, which increased, and was followed by other symptoms of enteritis. These were not combated by any proper remedies for a week, when he was first seen by Mr. Pakenham, who found him in a state of great exhaustion. Pulse 58, sharp and intermittent; countenance sunk; universal tenderness of the abdomen; frequent vomiting; diarrhœa and tenesmus: calomel and opium were exhibited, the belly was leeched, and the patient was placed in a warm bath. Next day, the pulse being stronger, he was bled; the blood was not inflammatory. He was admitted on the following day.

His countenance was then collapsed, anxious, and expressive of abdominal suffering; the extremities cold, and the pulse hardly perceptible. The respiration was hurried, and the abdomen swelled and exquisitely painful. Constant tenesmus.

Under all these circumstances, we determined on trying the effect of free doses of opium, having witnessed its good effects in the case of Clare, already detailed. Large doses of the black drop were given every second hour, and mercurial frictions used over the belly. On the next day a great improvement was perceptible: the pulse was full and soft; the extremities were warm; the face had lost the hipprocratic expression; he could bear pressure on the abdomen, and the tenesmus had ceased. The patient, who, on the day before, was nearly insensible to external objects, expressed great relief and confidence of recovery. The same treatment was persevered in for the next twenty-four hours, and on the following day *all symptoms of peritonitis had completely subsided.* The belly felt natural; there was no tenderness; the pulse was good, and the patient declared himself well. We now unfortunately changed our plan of treatment; the opium was omitted, and a gentle saline laxative exhibited; this produced four evacuations, followed by a return of all the symptoms of abdominal inflammation, under which he speedily sunk.

*Dissection, ten hours after death.*—The abdomen was distended and tympanitic; the intestines were every where agglutinated together, and adherent to the parietal peritoneum, except in the left iliac fossa, where a quantity of yellow puriform matter was collected. Small quantities of the same fluid were found between several of the intestinal folds, circumscribed by the effusion of lymph; general livid redness of the serous membrane. On detaching the caput coli from the peritoneum, lining the right iliac fossa, a small perforation of the gut was discovered by the escape of the contents of the intestine in a jet. The serous membrane lining the iliac fossa was found of a bluish color, and softened for a space of three square inches: this change was also observed in the lower portion of the ileum. On opening the gastro intestinal tube nothing remarkable was observed until within the last twelve inches of its termination, where the mucous membrane was softened, and dark colored. The perforation of the large intestine, before mentioned, was apparently the result of an apthous ulceration, as no glandular enlargement or ulceration could be detected round it. Here, however the mucous membrane was softened, and of a deep red color, while the remaining portion of the intestine was healthy.

2. *From Perforation of the Rectum.**—On Friday night, Jan. 10, 1834, at 11 o'clock, I was requested to visit Mr. J. M. Lee, of this city. On my arrival I found him in great agony, and the anxious expression of his countenance convinced me that he was extremely ill. He informed me, that he had been somewhat costive; and had, about 4 o'clock that afternoon, endeavored to procure an evacuation from his bowels, and during the effort, *he had ruptured some part.* He said he was conscious of it at the moment, and could not be mistaken—the same declaration he had already made several times to his family, whom he forbade to send for a physician, because he felt convinced that it would be unavailing.

He had been exposed at a late fire, and had taken cold, but his general health was better than it had been for months. I knew that his habits had been intemperate, and that he had labored under a violent attack during the first invasion of the cholera. I have since learned, that he had, until the last few weeks, been troubled with diarrhœa, but not to an extent, which, in his estimation, rendered a resort to medical aid necessary. Some few days before I saw him, it had given way to a torpid condition of the bowels. He had, in the early part of the day on which he was taken, been at work; and in conversation with his friends, he had frequently exulted in the prospect of returning health and vigor.

Immediately on the occurrence of what he termed a 'rupture,' the most excruciating pain commenced in the hypogastric region, and rapidly extended to the whole abdomen. The great rigidity of the muscles prevented distention; but the entire surface, as well as the deep seated parts, was so exquisitely sensitive, that the slightest touch produced an agony which made him cry out—indeed, his respiration was constantly interrupted by groans and screams.

* Communicated by John F. Henry, M. D.

My first impression, on hearing his statement, was, that he labored under strangulated hernia; and this opinion was strengthened by his peculiarly anxious countenance, and the soft and compressible character of his pulse; as well as by the fact, that two hours after the occurrence to which he ascribed his illness, he had vomited, and fainted. On examination, however, I was convinced that no such condition existed; and the best diagnosis I could form, was, that the peritoneum was in a high state of inflammation; but of the cause which could so suddenly develope it, I was unable to form any rational conjecture.

Under this view of his case, and with the hope that the inflammation might be prevented from terminating in mortification, to which it seemed to be rapidly tending, I ventured to take blood freely, although the pulse was but little accelerated, and in point of strength and volume, was below the standard of health. I prescribed, also, 40 grs. of calomel and two of opium, and hoped I would find him improved in the morning. Such, however, was far from being the result. On visiting him at that time, the pain was undiminished; he had slept none: and no evacuations from the bowels had taken place, notwithstanding the administration of the most stimulating enemata, during the latter part of the night. I then resorted to castor oil and epsom salts, alternately, with a continuance of the enemata. I drew blood a second time; and applied a blister, ten inches square, to the abdomen His stomach rejecting the oil and salts, and the injections failing to excite the bowels, I resumed the use of the calomel and opium; but it did not even *mitigate* his sufferings, although the proportion of the narcotic was large. On the next morning, about 11 o'clock, he expired having lived 43 hours from the commencement of his illness.

*Dissection.*—The case being one of great interest, as well as of much obscurity, I was anxious to ascertain the cause of its sudden accession and rapid progress, and obtained permission to examine the viscera of the abdomen. This was done 24 hours after death, and disclosed the following morbid appearances:

On opening the cavity of the abdomen, the peritoneal lining was found in a state of high inflammation, bordering on sphacelation. The greater omentum was highly inflamed, and reached down into the cavity of the pelvis, where it was attached by adhesions which seemed recent, as they were easily broken by the fingers. Appearances of chronic disease were visible, in the thickened and knobulated condition of this membrane. On lifting it up the whole peritoneal surface of the bowels was found inflamed, and covered with a purulent fluid, which gushed out whenever the adhesions which bound together all the convolutions of the bowels were broken. These adhesions extended throughout the whole course of the intestines, from the duodenum to the rectum; so that no one portion of the alimentary canal was loose and floating. The quantity of false membrane and pus was very great. On separating the bowels, spots of a dark livid color were found throughout their whole extent; but no part was in a state of actual mortification. There was, also, more than a quart, by computation, of an exceedingly offensive and feculent fluid; and on tracing the bowel, from the stomach to the rectum, an aperture was discovered in the anterior part of the superior portion of this bowel, large enough to admit the point of the finger. The mucous coat projected outward, as if its edges had been pressed through the opening by the escape of fluid or flatus. This, of course, was the cause of the inflammation and his speedy death; and explained, at once, the whole phenomena of this disease.

I regret that, from indisposition, I was compelled to desist from farther examination, as much light might have been thrown on the cause of this lesion, by examining the state of the mucous membrane. My conjecture at the time, was, that an ulcer of this membrane had gone on extending and deepening its cavity, until having almost destroyed the continuity of the parieties, the strong pressure of feculent matter and flatus, in the effort to procure an evacuation, had ruptured the remaining portion, and produced a communication between the cavity of the bowel and that of the peritoneum.

### IV. Escape of the Contents of the Gall Bladder.

### 1. *Softening of the Cystic Duct without Perforation.*

Before citing instances of bilious effusion, I will read to the Society the following case, from Dr. Abercrombie, page 384, as illustrating the manner in which perforations of the cystic duct *may* occur.

A lady, aged 60, had been for several years liable to attacks of acute pain in the right hypochondriac region, which generally continued in great severity for a few hours, and then subsided suddenly. On Wednesdsy, 14th January, 1824, she was seized with pain corresponding to her former attacks, but which did not subside as usual. It continued through the night, accompanied by frequent vomiting and constitutional disturbance. On the 15th, there was fever, with frequent vomiting and obstinate costiveness, and the pain was more extended,—being referred to a considerable space on the right side of the abdomen. Belly tense and rather tumid. The case had assumed the character of ileus, and all the usual means were employed with little relief.—16th. There was some discharge from the bowels after a tobacco injection, but it was very scanty. Severe pain continued, with every expression of intense suffering. Her strength sunk, and she died on the morning of the 17th.

*Inspection.*.—Every part of the intestinal canal was perfectly healthy, except the upper part of the duodenum, where there was considerable appearance of inflammation, with remarkable softening, so that it was very easily torn. A large irregular calculus was found sticking in the ductus communis, and the parts were so *softened that it came through the side of the duct when it was very slightly handled.* In the texture behind the duodenum there was considerable appearance of inflammation. No morbid appearance could be detected in any other organ.—*Abercrombie, pp.* 384–5.

It is evident, that if this patient had lived a little longer, the gall duct would have been perforated.

### 2. *Remarks on the escape of the Bile from Rupture and from Ulceration of the Gall Bladder or one of its Ducts.*

The immediate effect of this accident is rapid peritonitis, fatal in eighteen or twenty-four hours. The symptoms preceding it will depend upon its cause, and consequently may be either very obscure, or such as indicate great distention of the gall bladder, with obstruction of the bile in its passage out of it. The causes of the affection are chiefly referable to two classes.

(1st.) Obstruction of the common duct. This may take place rapidly by adhesive inflammation, or more slowly by gradual obliteration. In the former case the symptoms are rapid, as in a man mentioned by Andral, who had acute pain followed by jaundice, and a pyriform swelling rising up from under the margin of the ribs. On the 5th day he was suddenly attacked with peritonitis, and died in twenty-four hours. The ductus communis was found much contracted, and at one place obliterated. The gall bladder and the hepatic and cystic ducts bore marks of having been much distended; the rupture had taken place in the hepatic duct, and much bile was found in the peritoneal cavity. In another the symptoms of obstruction to the passage of the bile had been going on for between two and three months before the fatal attack, and in this case both the cystic and common ducts were found much contracted.

2d. Perforation of the coats of the gall bladder by ulceration. A man mentioned in the Noveau Journal de Medecine for 1821, had been affected for more than a month with pains in the abdomen and fever, which had various remissions and aggravations. On the 37th day of the disease, he was suddenly seized with symptoms of the most violent peritonitis, and died on the following morning after suffering inexpressible agony. On inspection, there were found marks of most extensive peritonitis. The inner surface of the gall bladder presented numerous small circular ulcers from one to three lines in diameter; two of them had entirely perforated its coats, so as to allow the escape of the bile into the peritoneal cavity.—*Abercrombie, pp.* 385 6.

3. *From Rupture.**—On Thursday, of the first week of September, 1829, I was called in haste to see Mr. Russel Bottsford. He complained of severe pain in the right hypochondriac region, attended with paroxysms apparently spasmodic, which caused him to draw down his breast towards the pelvis, and press his hands upon the upper part of the abdomen. He had vomited once or twice before I saw him.

He said he bad been in a profuse perspiration the evening before, and had retired to bed early, in a very open counting room. The weather had become suddenly cold during the night, and he awoke about 4 o'clock in great pain and distress.

I had seen him in several attacks of a very similar kind, during the two years preceding, which had yielded to the prompt administration of an anodyne and cathartic dose, together with hot applications to the epigastrium.

It was now 9 o'clock, A. M. He had been very chilly before I saw him, but a considerable reaction had taken place; his pulse was somewhat tense, and his manner agitated, showing much disturbance in the nervous functions. He was immediately bled, but the blood did not run freely, so that not more than 10 ounces was taken. I then administered calo-

* Communicated by the attending Physician.

mel and pulvis Doveri, each 15 grs.; had a large mustard poultice applied over the abdomen, and ordered the best means to be used to encourage perspiration.

At 2 P. M. the pain was somewhat abated, though still attended with paroxysms of considerable severity. He was now bled freely, a large blister was applied over the seat of the distress, and more active cathartics were administered.

At candlelight I saw him again. The pain was less severe; only one slight operation from the bowels; skin dry.

About 10 o'clock the same evening, he had severe paroxysms of pain, attended with violent vomiting, after which the pain left him. During this violent paroxysm Dr. W. was called in; but as the pain had abated no new treatment was ordered.

On Friday he was comparatively easy; had two small evacuations from the bowels; skin dry, but no fever; and he considered himself as getting well.

On Saturday morning he told me he was better, and should be out in a day or two. His pulse was feeble, and he complained of being weak, the skin was cool, and softer than yesterday.

About noon he grew worse. Dr. W. saw him, with me, soon afterwards. He was evidently sinking; the pulse was 120, weak and small; the extremities livid, and inclining to be cold; and the eyes glaring. Ordered him to be sponged freely with the nitro-muriatic wash as hot as could be borne, and sinapisms to the extremities.

He passed a restless night, complained of great oppression; an undefinable feeling in the epigastrium, and great heat along the spine. His mental faculties were not impaired, and he apprehended no danger until he was informed he was dying. He expired Sabbath morning, about 11 o'clock, 78 hours after the attack.

*Dissection.*—Leave being obtained, the body was examined by Dr. C. and myself, 24 hours after death.

On opening the abdomen, a large quantity of ropy greenish-yellow fluid, with whitish, curdly looking matter floating in it, was diffused throughout its cavity.

The bowels were greatly distended; no recent inflammation or increased vascularity appeared in any of the abdominal viscera.

There were marks of his having suffered a great degree of inflammation in the hepatic region, at some former time: viz: the liver adhered firmly to the abdominal parieties throughout its whole extent, the anterior edge of its lower surface adhered closely to all the organs on which it naturally rests. The peritoneum was greatly thickened, and many adhesions existed between the organs in the neighborhood of the liver.

On detaching that organ carefully from its natural adhesions, the gall bladder (which seemed to have been unusually large) was flaccid, with a rent in its side, into which the finger might be thrust. It contained a calculus three-eighths of an inch in diameter, with a very rough (mulberry) surface. No other appearance of disease was discovered.

Should it be asked, "at what period I suppose the rupture to have taken place?" I would say, in answer, that I believe it to have happened on Thursday night, in the violent fit of vomiting which then occurred, and after which the pain or spasm left him; about 18 hours from the first attack, and 60 hours before death.

4. *From Perforation of the Cystic Duct.—Case of John B. Brooke, of Cincinnati.* This gentleman was about 34 years of age. He had experienced more than one attack of torpor and deranged functional action of the liver. His temperament was nervous, and his state of mind, under all kinds of indisposition, alarmed and hysterical.

On the evening of Saturday, January 18, 1834, being in his usual state of health, after having taken no dinner, and eaten an ordinary supper, he was seized with acute pain in the right side, about the region of the liver. The pain abated in a short time, and he walked out; but about 12 o'clock at night it returned with excruciating violence, and was then chiefly in the lower or hypogastric portions of the abdomen. Dr. Robert Moorhead was then called in, and soon afterwards Dr. John Moorhead. The next morning he was costive, and though easier, by no means free from pain, and continued in that way (with an occasional exacerbation) for the next 24 hours. His abdomen was swollen, and tender on pressure, and he was thirsty. On Monday morning, having slept none through the night, he was still worse. In moving he felt something give way, as he thought, within him. His costiveness still continued. About noon he began to vomit. In the evening, Dr. Eberle was called into consultation. On Tuesday morning these gentlemen were *replaced* by Dr. Richards and myself. We first saw the patient about 9 o'clock. He was then using a mixture of castor oil, spirits of turpentine, and croton oil; with stimulating injections every half hour or oftener, which had already been repeated a great number of times. A large blistering plaster covered the anterior part of the abdomen. He had taken assafœtida, cathartic pills, infusion of senna, pills of croton oil; and was then on the use of the mixture just mentioned. On the evening before, he had been bled to about 14 ounces. The blood exhibited considerable siziness.

When we first saw him, the following were the symptoms: sleepiness from the beginning; countenance, haggard and pale; dyspnœa, very great; posture, on his back, and unable to turn on either side; abdomen, from the epigastrium to the pelvis, swollen, tense, still extremely painful, especially near the symphysis pubis, and exquisitely sensible to pressure; constipation, still remaining; heat of the extremities reduced; pulse 124, weak and small; secrction of urine suspended, as was ascertained by introducing the catheter, when the bladder was found empty. The whole of these symptoms continued except the pain and soreness, which, gradually abating, ceased altogether by 3 or 4 in the afternoon, when he declared himself better, and said if he could get some sleep he would soon be well. But his pulse was then imperceptible, and he expired between 5 and 6, without delirium.

Soon after being called in, I was struck with the resemblance of the symptoms of this patient to those of E. P. and J. McD. just read to the Society from the Western Journal, and mentioned it to Dr. Richards, and some of the friends of the patient, inquiring of the latter, whether Mr. B. on the day previous to his attack, had suffered any violence, or made any great and sudden effort, that could have ruptured the stomach or bowels, but was assured he had not.

On analysing the present and the past symptoms, we came to the conclusion, that it was a case of peritonitis, about to terminate in gangrene, and expressed this opinion to the friends.

There seemed little or no prospect of saving him, but as he and his family insisted on our attempting something, we gave him two or three doses of calomel in quick succession, and resorted to cupping. This was undertaken, first, over the epigastrium, where the blister had acted as a rubefacient, and afterwards on the sound skin of the flank. His sensibility, both to the scarification and the cups, was acute, but the skin was so exsanguinous that very little blood was obtained. During the operation, however, and subsequently, he had several small and liquid alvine evacuations, but they were not followed by any relief. By discontinuing all kinds of drink, his vomiting ceased. Warm fomentations were applied to the abdomen, and in the afternoon he took two moderate doses of laudanum. This completed the treatment of the case.

*Post-mortem Examination.*—The next morning the Doctors Moorhead obtained permission to examine the body. Dr. Richards and myself were not requested to attend; but a friend of that gentleman invited him in. The following were the appearances, as reported by Dr. Smith:

"The cavity of the abdomen contained a quart or more of a ropy gelatinous fluid, of a bilious color. The peritoneum was generally tinged with a bilious deposition; and where it covers the right lobe of the liver and the diaphragm, the deposition was thicker than elsewhere. Over these parts the fluid seems to have been retained some time before its general effusion throughout the abdominal cavity. There were some slight adhesions, which were not of recent formation, between the upper edge of the omentum and the liver, just over the cystic and hepatic ducts. The gall bladder was contracted, and there was near its neck, a small opening, arising from ulcerations, probably caused by a rough gall-stone, which occupied the ulcerated cavity.

The descending arch of the colon, the sigmoid flexture, and the rectum, were contracted to less than an inch in diameter. All the other parts of the colon, also the ileum, and part of the jejunum, were very much distended, and were filled with air.—Several coils of the ileum, next the caput cœcum, of SOME FEET IN LENGTH, were lying deep in the pelvis, between the bladder and rectum. The mucous coat of the stomach and bowels presented no unnatural appearance. There were no traces of inflammation hyperæmia, or mortification. The other viscera presented a natural aspect. No further examination was made."

Such were the symptoms, and morbid appearances, in the case of Mr. Brooke, who seems, manifestly, to have died from the impress of bile upon his peritoneum. No treatment did him any good, and no treatment could have saved him. I shall trouble the Society no further.

---

### APPENDIX.

I propose to add something to the paper read before the Society. It cannot, I think, be found uninteresting to the readers of the Journal, as it will involve several questions in pathology and morbid anatomy.

A few days after the death of Mr. Brooke, the Doctors Moorhead brought his case before the public, in the common newspapers, and in hand-bills distributed through the city and among the students of the Medical College of Ohio. In their several publications, which abounded in libels on my professional and private character, and in a manuscript communication to the Cincinnati Medical Society, where, *subsequently* to their first publication, I gave an account of Mr. Brooke's case, they presented the following points:

1. That Mr. Brooke died from *Colic*, and not from the escape of bile into the cavity of the peritoneum, and that inflation of the bowels is evidence of previous colic.

2. That during his illness, they considered that his colic was dependent on intussusception or on displacement of the bowel.

3. That three feet of the ileum was lodged in the pelvis, between the bladder and rectum, both of which were empty, and that this *displacement* was the cause of the colic which produced his death.

4. That a rupture of the gall bladder is not necessarily fatal; and that a case is on record, in a book of high authority, which was cured.

5. That the rupture in Mr. Brooke's case did not take place till the day preceding his death.

6. That the cupping, practised in the forenoon of the day on which he died, at sun-down, was a cruel and unjustifiable measure.

7. That there was no inflammation.

I am almost ashamed to fill up the pages of this Journal with comments on these different propositions; but the distant reader will perhaps excuse me, when he is informed, that one of the gentlemen has for many years been at the head of the Medical College of Ohio, and regarded by its trustees, as one of the ablest physicians in America. The published pathological and therapeutic opinions of *such* an official, may not be authoritative with his *pupils*, but may have weight with physicians who, living at a *distance*, cherish respect for the distinguished faculty to which he belongs, and are, therefore, entitled to notice. I shall examine them *seriatim*.

1. Mr. B., it is asserted, died of *Colic*. The stress laid on this word by the professor, would seem to indicate, that he regards colic as a disease not less specific than the small pox or hooping-cough. In his adherence to the antiquated nosology of the school in which he was educated, he seems to be unaware, that the word colic is vaguely applied to a group of symptoms, consisting of intermittent abdominal pain, constipation and swelling and that these symptoms may arise from a great variety of lesions, and be, or not be, accompanied by inflammation. In the case of Mr. B. what produced the colic—that is, the pain, inflation, and costiveness? On the day previous to his attack in the evening, he was in his usual health, and did nothing, suffered nothing, ate nothing, drank nothing, that could have thrown the muscular tunic of his bowels into morbid action. The attack was sudden and violent, and unproduced by any obvious cause.

But the Professor seems to regard an inflated state of the bowels, as characteristic of *colic*, and consequently absent, after death from any other disease! Who has ever made *post mortem* examinations, without meeting with inflation of the bowels, when none of the symptoms of colic existed during life? Who has not witnessed the abdominal distention, which precedes death from the bilious fevers of the West? Who does not know, that acute peritonitis, when it proves fatal, is attended with abdominal distention? As a specimen of this, I will quote the following cases from Cooke's Morgagni:

"A young man, twenty-two years of age, was wounded in his groin by the horn of a cow. He had fever, and by degrees his face and the remainder of the body began to swell, but the swelling was not strictly œdematous. Besides this, nothing of importance resulted till the seventeenth day, when respiration became difficult, and these affections were accompanied with pain, and the sensation of a bolus occupying the fauces. He was also occasionally agitated by tremor, and the tumefaction of the body not only continued but increased. The swelling which occupied the groin extended, and he died about the twenty-second day.

*Dissection.*—The wound began near the abdominal ring, and extended between the rectus muscle and the tendons of the obliqui; and in this space a considerable quantity of coagulated blood was found. *The intestines were greatly inflated;* there was a copious redundance of serum in the abdominal cavity, and a similar effusion had taken place into the thorax."—*Valsalva, liv.* 2.

"A foreigner received a wound from a thin two-edged sword which penetrated the abdomen obliquely, on the left side, beneath the ribs. The narrowness and obliquity of the wound were such that even a probe could not be introduced. From the time of receiving the wound till the fifth day, (on which he died) he vomited bile, with the ingesta. He was tortured with pains in the belly, and the intestines never acted except by means of stimulating clysters. Towards the close of life blood escaped from the mouth and nostrils.

*Dissection.*—*The intestines were distended with gas,* and beneath them a small quantity of blood was extravasated. The sword had reached to the intestinum colon, about four digits below the spleen; but it had injured it only superficially. The weapon then passed onward and wounded the mesentery, which was also found swollen.

*Morgagni, liv.* 35.

2. The Professor informs us, that during the illness of Mr. B. he believed that he labored under intussusception, or displacement of the bowel, and that his colic was occasioned by that anatomical lesion. If either of these accidents had existed, what was the appropriate treatment? For myself, I answer, large doses of opium, tobacco injections, blood-letting, cupping, the warm bath, copious enemata of tepid water, and finally, mild cathartics. What was the treatment pursued under the theory of that anatomical casualty?—cathartic pills, pills of croton oil, senna tea, a mixture of castor oil, oil of turpentine, and croton oil, incessant, irritating injections, a large blister, and one bleeding not long before the death of the patient, to "*prevent* inflammation." I would ask how these drastics were to restore an invaginated or a displaced bowel? Would they allay spasm, or blunt morbid sensibility, and assuage pain, or obviate inflammation? If invagination or displacement existed, would any harm be done, if inflammation did not supervene? Who ever heard of death from either of those affections, but by the coming on of inflammation; and who would expect to ward off that condition, by the unrelenting and unmixed administration of irritating cathartics?

3. That not less than three feet of inflated ileum was lodged in the pelvis, and that this was a displacement, and the cause of the colic, which occasioned death, is rather a startling enunciation to come from a learned Professor. I shall not inquire how *three feet* of *inflated* bowel could be condensed into the male pelvis! Dr. Richards, who was present at the dissection, supposed that there were 12 or 15 inches. But whether more or less, it is agreed by all, that the bowel was not invaginated, strangulated, or inflamed; how then could its presence in the pelvis, even if that constituted a displacement, occasion the death of the patient? But what shall we think of a Professor, who considers a portion of the small intestines *out of place*, and the cause of death, when he finds it in the *pelvis*—the bladder and rectum both being empty, and contracted to their smallest dimensions? When the organs do not fill up the pelvis, what is there to prevent a part of the small intestines from sinking down into that cavity? Whenever the abdominal parietes are cut through, do not the contents of the abdomen issue forth, and even sometimes hang down to the patient's knees? Has not the late Mr. John Bell written a chapter to show, that the contraction of the abdominal muscles, keeps the bowels at all times so compressed, that even the air of the atmosphere, when the former are wounded, cannot make its way into the cavity of the peritoneum? And does not this compression constantly cooperate with gravity, in causing a portion of the ileum to rest upon the fundus of the bladder and the anterior surface of the rectum? On this point, what say the common systems of Anatomy? Let us make a few quotations from the first works that present themselves:

"The rest of the small intestines having no certain seat or division, is continued by innumerable and uncertain convolutions, not to be described, so as fill out the lower part of the abdomen and the *pelvis*." —*Haller*.

"The ilium is the continuation of the jejunum, situated in the hypogastrium, and *very often* some part of it in the *pelvis*."—*Chesselden*.

"When the viscera of the pelvis are empty, a *large* portion of the small intestine is in the *pelvis*."—*Wistar*.

"The pelvis, as a whole, is a conoidal cavity, having its base upwards and the summit below. Its internal surface forms an irregular floor, on which the *viscera of the abdomen are sustained* in the erect position."—*Horner*.

"The pelvis is a large, irregular, bony cavity, open above and below, which terminates the trunk beneath, and supports or contains part of the *intestines*, &c."—*Cloquet*.

Thus we see, that the Doctor has mistaken the *natural and constant* position of a portion of the small intestines for a *displacement*, and deduced from it a *mortal Colic*.

If these quotations expose the ignorance, in simple descriptive Anatomy, of a cherished professor, and thus cast a stigma on the institution of which he is one of the luminaries, the fault is not mine but his; for he gratuitously thrust himself, and rudely dragged me, before the public; and if the exposure which I have made, should give offence to the state of Ohio, which is fostering him for the benefit of her sons, I shall regret it; but will say, as their friend, that, if what I have written should prove a stimulus to his improvement, and secure better teachings for them in future, I shall have something to solace me under her censure.

4. That an escape of the bile from rupture or ulceration of the gall bladder, is not of necessity, a fatal event, certainly requires proof. To say that there is a case on record, which terminated favorably, is rather a singular assertion. I would ask, if the patient did not die, how it could be known, that his gall bladder was ruptured? No *student* of Medicine could confound the effects of a *wound* of the gall bladder, with those of its rupture from distention, or the escape of its contents by a perforating ulcer. In the first case, the wound might happen when the cyst contained but little bile, and heal up by the first intention, thus preventing a further flow, even after a few hours, but nature has no such salutary resource, when the parieties are torn or eaten through by ulceration. The bile then continues to flow, and must always prove fatal. Even simple punctured or incised wounds of that organ, are generally followed by death. The instances of recovery are but exceptions to the rule: in cases of rupture and ulceration, there can be no exception to the law of a fatal result.

5. Another gratuitous assertion, is that the rupture in Mr. Brooke's case did not occur till the day before his death. The symptoms appear to me to call for a very different conclusion. The patient was in his usual health, when suddenly seized, without any obvious cause, with acute pain in the *region of the liver*, not the umbilicus, as in colic. This pain, obviously, arose from the first, perhaps a moderate, escape of the bile into the cavity of the peritoneum. He walked about, and in a few hours, the pain was tranferred to the hypogastric region, where the bile would of course gravitate. From this time forward his sufferings were acute, his bowels costive, and his abdomen swollen and tender under external pressure. Thirty-six hours afterwards, when he was rising up in bed, another discharge seems to have taken place. The opening in the neck of the gall bladder, was no doubt enlarged, and the cyst then probably emptied itself. No new symptoms arose, but the old became more intense. Thus the whole history of the case indicates, that all the symptoms came from the progressive escape of bile into the peritoneum, and its irritating action upon that membrane.

6. That the cupping which I attempted, was unjustifiable, because the patient died in six or seven hours afterwards. Suppose he had died in one, what harm would the cupping have done? If he had been *in articulo mortis*, it could not have been felt, and would not, therefore, have increased his sufferings: if his sensibilities were still so acute, that the application of the scarificator gave him pain, a well founded hope might be cherished, of its doing him good, provided he labored under inflammation. The fact is, that he was not in the jaws of death, nor insensible; but took an active interest in the operation, and complained in the usual degree, not only of the sarification, but of the pressure of the cups. I would put it to the profession to say, whether in peritonial inflammation, when general bleeding is no longer admissible, cupping and leeching, are not our most precious resources; and whether any physician would be justifiable in omitting them, on account of the pain they might give the patient? On this point, I believe there can be no difference of opinion.

7. Lastly, it has been affirmed with great confidence, that in Mr. Brooke's case there was no inflammation. An inquiry into this matter cannot be unprofitable. 1. The authorities cited in the paper which I read before the Medical Society, establish the fact, that the escape of bile into the peritoneum, generally excites inflammation; though, in a less degree, I believe, than an escape of the contents of the stomach and bowels. Now in Mr. Brooke's case, it is admitted, that there *was* an effusion of bile upon that membrane. And a cause of inflammation therefore existed. 2. The gall bladder was so contracted, as to be buried in the liver; and it is not probable, therefore, that it contained much bile at the time when the perforation of its neck occurred. If so, whence came the *quart* of ropy fluid, which the peritoneum contained? Undoubtedly, in a great degree, from the surface of that membrane, indicating an increased secretory action, which carried off the turgescence of the capillary vessels. 3. The surface of the bowels, the liver, and the diaphragm, was covered with a "bilious deposite," which according to Dr. Smith, was thickest on the *under* side of the last organ. But if it had been a deposite, it should have been thinner there, for deposites cannot be made upwards. I have no doubt that the "bilious deposite," was an exudation from the surface of the peritoneum, colored by the bile, and a consequence of inflammatory action. 4. If there was no inflammation, what produced the size on the surface of the blood drawn the night before the patient's death? 5. What could create such great tenderness of his abdomen, (rendering pressure intolerable,) but inflammation? 6. The absence of a loaded state of the capillaries after death, is not conclusive evidence that inflammation had not existed. On this point many authorities might be cited, but shall content myself with one. Dr. Eberle (*Theory and Practice of Medicine, Vol. II.*) after speaking of the inflammatory nature of Anasarca, observes:—

"Subacute inflammation of this membrane, (the peritoneum,) in whatever way it may be produced, terminates perhaps always in effusion; although in some instances, this may not be so copious as to constitute dropsy. In the majority of fatal cases of ascites, the peritoneum exhibits a highly injected state; and in many instances, the traces of previous inflammation are still more conspicious and unequivocal, its structure being either thickened and otherwise altered, or covered with an infinitude of miliary tubercles. Occasionally, indeed, no marks of pre-existing inflammation whatever are to be seen; but the investigations of modern pathologists have rendered it abundantly manifest, that where no disorganization or structural change has been effected, the mere redness, or injected state of inflamed parts, may and does often disappear in *articulo mortis*, or even *post mortem*."—*p.* 429.

From these facts and considerations, I do not hesitate to express the opinion, that inflammation did take place in this case, but do not believe it was very acute. The temperament of the patient was eminently the *nervous*, and the disease which was awakened in the peritoneum partook largely of the character of irritation. But the question relative to Mr. B. is *not* whether he died from inflammation of the peritoneum, or from irritation of that membrane. The inquiry is this—Did he die from a disease of the peritoneum occasioned by an effusion of bile, or from colic produced by the lodgement of a portion of the small intestines in the pelvis, its natural situation? That it was from the former, seems to me absolutely incontrovertable; and such was the opinion expressed by Drs. Smith and Eberle, immediately after the *post mortem* examination was made.

*March* 1*st*, 1834.

